# Efficient identification of CRISPR/Cas9-induced insertions/deletions by direct germline screening in zebrafish

**DOI:** 10.1186/s12864-016-2563-z

**Published:** 2016-03-24

**Authors:** Isabel Brocal, Richard J. White, Christopher M. Dooley, Samantha N. Carruthers, Richard Clark, Amanda Hall, Elisabeth M. Busch-Nentwich, Derek L. Stemple, Ross N. W. Kettleborough

**Affiliations:** Wellcome Trust Sanger Institute, Wellcome Trust Genome Campus, Hinxton, Cambridge, CB10 1SA UK; Twist Bioscience, 455 Mission Bay Boulevard South, San Francisco, CA 94158 United States

**Keywords:** CRISPR, Cas9, Zebrafish, Germline, Genome editing, Perl, Mutagenesis, Embryo, Next generation sequencing

## Abstract

**Background:**

The CRISPR/Cas9 system is a prokaryotic immune system that infers resistance to foreign genetic material and is a sort of 'adaptive immunity'. It has been adapted to enable high throughput genome editing and has revolutionised the generation of targeted mutations.

**Results:**

We have developed a scalable analysis pipeline to identify CRISPR/Cas9 induced mutations in hundreds of samples using next generation sequencing (NGS) of amplicons. We have used this system to investigate the best way to screen mosaic Zebrafish founder individuals for germline transmission of induced mutations. Screening sperm samples from potential founders provides much better information on germline transmission rates and crucially the sequence of the particular insertions/deletions (indels) that will be transmitted. This enables us to combine screening with archiving to create a library of cryopreserved samples carrying known mutations. It also allows us to design efficient genotyping assays, making identifying F1 carriers straightforward.

**Conclusions:**

The methods described will streamline the production of large numbers of knockout alleles in selected genes for phenotypic analysis, complementing existing efforts using random mutagenesis.

**Electronic supplementary material:**

The online version of this article (doi:10.1186/s12864-016-2563-z) contains supplementary material, which is available to authorized users.

## Background

The CRISPR/Cas9 system has emerged over recent years to become the prevailing method for genome editing with many different applications in a wide variety of organisms [1–11]. Clustered Regularly Interspaced Short Palindromic Repeats (CRISPR) are sequences found in many species of bacteria and archaea, consisting of repeated sequences interspersed with different non-repetitive sequences. These spacer sequences are derived from previous viral infections and the CRISPR/Cas system functions as an adaptive immune response to viral infection [12–15]. CRISPR associated (Cas) genes are arranged in operons next to CRISPR loci [16].

The endogenous type II CRISPR system is composed of a CRISPR RNA (crRNA) derived from the spacer sequences [17], a trans-activating RNA (tracrRNA; [18]) and the Cas9 endonuclease, which cuts at sequences complementary to the crRNA [19]. The form most commonly used in genome editing applications employs a single chimeric version of crRNA and tracrRNA known as a synthetic guide RNA (sgRNA). This can be used to efficiently induce small insertions/deletions (indels), through inaccurate repair of double-strand breaks, preferably generating frameshift mutations to produce loss-of-function alleles for genes of interest. Cas9 protein recognises a small motif known as the proto-spacer adjacent motif (PAM), which is present in the foreign sequence, but not in the CRISPR locus, allowing it to distinguish self versus non-self [20–23]. For *Streptococcus pyogenes* Cas9, the PAM sequence is NGG, leading to a consensus CRISPR target site of N21GG. For sgRNAs produced *in vitro* using a T7 promoter the first two transcribed bases are G, making sgRNAs with a GGN19GG consensus [24, 25].

The CRISPR/Cas system has made it feasible to generate targeted mutations on a large scale. Previous approaches relied on either chemical or insertional mutagenesis [26–28], and while these were very successful they suffer from a problem of diminishing returns, because of their random nature. Also, genes that are haploinsufficient will not be recovered by these strategies, as F1s with such alleles will not survive to be screened. The ability to disrupt gene function in a targeted high-throughput manner provides a complementary approach with the potential to realise the goal of producing a knockout allele in every gene in the zebrafish genome. Beyond that it will also make other genomic modifications straightforward, enabling the study of non-coding portions of the genome such as non-coding RNAs and enhancers.

The most time-consuming aspects of generating mutants by CRISPR/Cas are screening and genotyping. For example, sgRNAs need to be screened for cutting efficiency as there is currently no reliable way of predicting the activity of any given sgRNA. Mosaic sgRNA-injected individuals then need to be screened for those that transmit the appropriate alleles (e.g. frameshift indels or missense mutations). Finally, individuals need to be genotyped to identify those that are carrying such alleles. Capillary sequencing is expensisve and not high throughput and methods such as T7 endonuclease I assay and High-Resolution Melt Analysis do not identify which alleles are useful. Since G0-injected individuals are mosaic for induced indels, there is also the problem of what tissue to screen to best evaluate germline transmission rates.

We have developed a scalable design and analysis pipeline for the production of CRISPR/Cas9 mutants. To streamline screening, we use high-throughput next generation amplicon sequencing of sperm samples from G0-injected males. This allows us to cryopreserve sperm samples at the same time, producing an archive of alleles. It also provides information on the specific variants in the germline of each individual enabling us to design genotyping assays to the particular variants in each sample. This greatly simplifies the identification of F1 carriers and allows us to segregate multiple different alleles transmitted by the same founder.

## Results and discussion

### CRISPR/Cas9 sgRNA design

There are currently many web sites and programs for designing sgRNAs and determining possible off-target sites (see [29, 30] for summary) all with differing strengths and weaknesses depending on the particular application. For example, only a few allow design of sgRNAs in batch. To facilitate the design of sgRNAs for large numbers of target genes, we have created a set of Perl modules and scripts (https://github.com/richysix/Crispr). The modules define objects that represent components involved in the design process such as a target region of DNA, a CRISPR/Cas9 target site and PCR primers. The scripts automate particular parts of the process such as scoring CRISPR target sites and designing PCR primers for amplicon sequencing. Since there is currently no reliable way of predicting the cutting efficiency of a given sgRNA, targets are selected to minimize possible off-target effects.

**Fig. 1:**
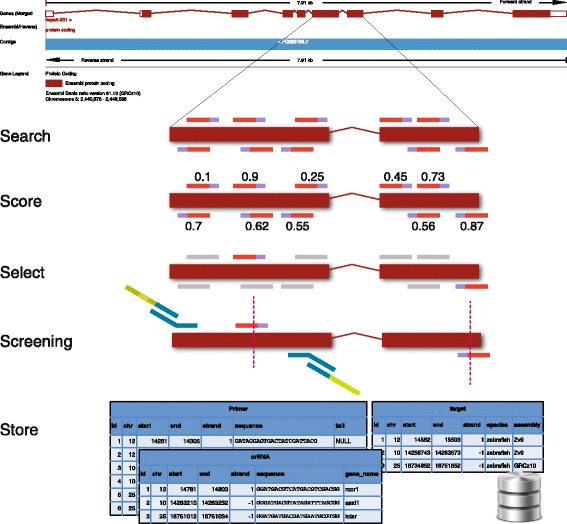
CRISPR sgRNA design process. An Ensembl gene model is shown at the top; two exons are expanded in the rest of the diagram. The target sequence is searched for CRISPR sites, these are scored and the best scoring are selected. For screening, PCR primers are designed for making amplicons into sequencing-ready libraries. The package also includes a database schema for storing information on CRISPR designs and screening information

The design process is illustrated in Fig. 1. The design scripts take either Ensembl identifiers (gene, transcript or exon) or genomic regions as input. Allowing arbitrary regions as input enables design of sgRNAs to modify non-coding regions such as promoters and enhancers. In the search phase, the sequence is scanned for all possible sites matching a given target sequence (e.g. GGN19GG). These sites are scored for off-target potential. Currently, off-target scoring is done by aligning target sequences back to the reference genome using bwa, allowing an optional number of mismatches. However, faster algorithms for off-target finding exist [29, 31] and these will be incorporated into the design module. All the possible target sites are output with a score that reflects both off-target potential and, if using Ensembl identifiers, the position of the target site in the supplied gene/transcript. Optionally, sites can be filtered based on known SNP data to avoid possible mismatches to the target site in the line being used.

Once CRISPR target sites have been selected, PCR primers for screening sgRNA efficiency can be designed using another script that runs Primer3 [32, 33] multiple times to produce nested primer pairs. The Crispr package also contains a SQL (MySQL/SQLite) database schema that holds information about target regions, CRISPR sites, construction oligos, PCR primers etc. There are also scripts to automatically load the information into a database using the output files from the design scripts.

For this study, sgRNAs were designed as pairs to allow larger than normal deletions to be made. In addition, these pairs could be used with Cas9 nickase [7] rather than native Cas9 if off-target effects proved to be a significant issue. The criteria for picking pairs were as follows: pairs of sites were considered valid if the predicted cut sites were between 30 and 100 bp apart and the CRISPR sites were in a tail—tail orientation (i.e. the first site targets the reverse strand and the second targets the forward strand). Evidence from human cells showed that such an orientation was much more efficient than head—head when using Cas9 nickase [7]. The usual target sequence when designing sgRNAs for *in vitro* transcription is GGN19GG to incorporate the end of the T7 promoter sequence [5, 25]. However, this makes finding correctly spaced and oriented sites for designing pairs of sgRNAs much less likely. To allow us to design pairs, we relaxed the sequence constraint to N21GG and placed two extra G nucleotides on the 5′ end, thus making the sgRNA 2 bases longer. Each pair of sgRNAs was injected together using native Cas9 and screened for efficiency using a single amplicon spanning both CRISPR sites. All the target sequences for the sgRNAs used in this study are in Additional file 1.

### Generating mutants using CRISPR/Cas9

**Fig. 2:**
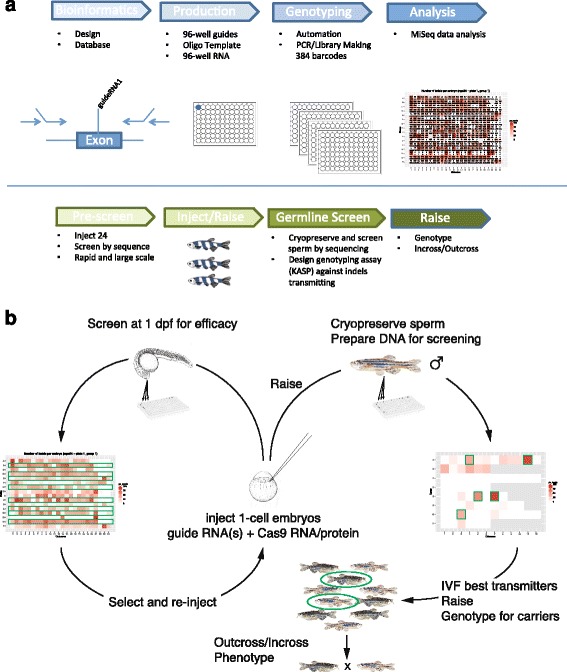
CRISPR workflow. **a** Overall workflow. Diagram showing the steps of the process. **b** Strategy for sgRNA screening. Initially, sgRNAs are screened for efficiency and those with high cutting efficiency are re-injected. The G0 embryos are raised and males are screened for germline CRISPR-induced indels. For high-transmitting samples, embryos are generated by IVF, raised and the resulting F1 carriers are identified by KASP

An illustration of our screening workflow for generating mutants using the CRISPR/Cas9 system is shown in Fig. 2. First, sgRNAs are designed by selecting target sites with low off-target potential in the region of interest. Generally, we find that selecting two target sites per gene is enough to find at least one sgRNA that is sufficiently active. RNA for microinjection is produced *in vitro* using the method of Gagnon et al. [5], which can be done in 96 well plates without any cloning steps.

To screen the activity of sgRNAs in a high-throughput manner, we use PCR primers designed for use with the MiSeq sequencing platform to amplify the region surrounding the CRISPR target site. This allows us to screen amplicons in hundreds to thousands of embryos in a single sequencing run. Typically, we first assess sgRNAs by injecting small numbers of embryos and screening for cutting efficiency by amplicon sequencing (Fig. 2b). Selected sgRNAs that efficiently induce indels are re-injected and the embryos raised to adulthood. Males are selected from these families and sperm is collected for both cryopreservation and screening for germline mutations, again by sequencing. The samples that carry high frequency frameshift alleles are selected for *in vitro* fertilisation (IVF) to produce F1 families. Screening the germline of G0 founder fish allows us to design KASP genotyping assays (LGC Genomics) for the specific variants that will be transmitted to the next generation enabling us to quickly identify F1 carriers for incrossing and subsequent phenotyping.

### Screening the germline by amplicon sequencing

The second part of the process of producing mutant lines is identifying CRISPR-induced indels, for both initial efficiency testing and recovery of transmitting alleles. Like many groups [34–40], we have used high-throughput sequencing of amplicons for identifying variants. The Illumina MiSeq platform allows fast turnaround times and provides enough reads to screen hundreds of samples over many amplicons. Nested primers are designed to amplify a 250–300 base pair (bp) region surrounding the CRISPR target site. The internal primers have partial Illumina adaptor sequence to allow for the creation of sequencing-ready libraries by PCR. Amplicons from a single sample (embryo/sperm sample) are barcoded using primers containing Illumina adaptor sequence and an 11 bp barcode (Fig. 1).

**Fig. 3:**
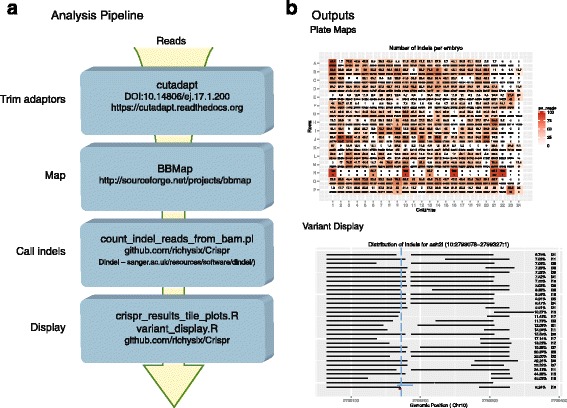
Crispr analysis pipeline. **a** Analysis pipeline. Schematic of sequence analysis procedure. **b** Pipeline outputs. Examples of the visualisations that the pipeline produces. Top—plate map showing the percentage of reads containing an indel for each sample along with the total number of reads. Bottom—display of induced variants relative to the CRISPR target site

The analysis procedure is shown in Fig. 3a. First, adaptor and primer sequence is trimmed from the reads using cutadapt [41]. Large deletions within the amplicon will result in the sequence reading into the adaptor sequence on the other side of the amplicon. Primer sequences are also trimmed to reduce the possibility of false positives caused by primer-dimer contamination. The trimmed reads are then mapped to the genome with the BBMap aligner (sourceforge.net/projects/bbmap/). BBMap is better able to map reads containing large indels which is essential for this application.

These alignments are then analysed by a custom Perl script that selects candidate alleles from the BBMap-produced BAM files and downsamples the reads into separate allele-specific BAM files [42]. These are analysed using the program Dindel [43] to call the specific insertions/deletions. The script then outputs each allele found and its frequency within the sample. Two custom R scripts are used to produce graphical representations of the data. Examples of these are shown in Fig. 3b. More detail on how to run the design and analysis scripts is provided in Additional file 2 and in the Crispr repository.

### Screening somatic tissues versus the germline

To investigate the best way to screen potential founder G0 individuals we compared the CRISPR-induced alleles present in the germline and the soma. We isolated both sperm and fin tissue from individual males from CRISPR-injected families and analysed them by amplicon sequencing. We tested nine different sgRNAs for five different genes.

The questions we were interested in were:

1. Is the percentage of reads showing an indel in the fin clip predictive of the percentage of reads showing an indel in the germline?
2. Do the variants found in the fin clip reflect the ones in the germline?

**Fig. 4:**
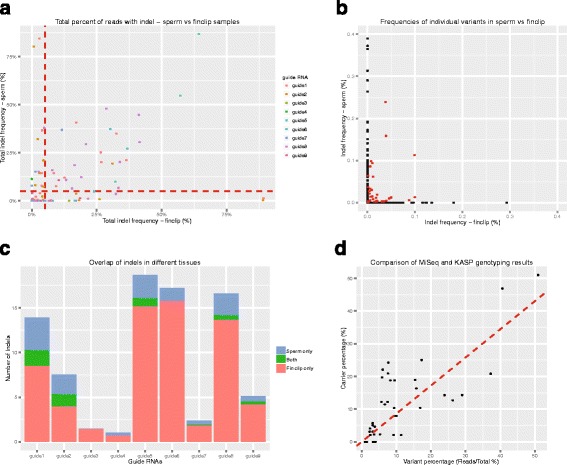
Comparison of induced indels between germline and somatic tissues. **a** Plot of total percentage of reads with an indel in sperm versus fin clip. **b** Plot of frequencies for individual variants in sperm versus fin clip. Variants that are present in both the sperm and fin-clip samples from a single individual are in red. Plot has been cropped to make points near the origin easier to distinguish. Figure S2 is the original. **c** Plot showing the average overlap of indels in each tissue for each sgRNA. **d** Plot showing the correlation between the frequency of reads from MiSeq data and the number of carriers identified in F1 outcrosses

As shown in Fig. 4. the complement of alleles found differs greatly between the two samples. Overall, there is a correlation between the percentage of reads with an indel in each tissue (Fig. 4a), although there are many clear examples where this is not the case (i.e. a fin clip with a high percentage where the sperm sample has a low percentage and vice versa). The correlation between the percentage of reads showing an indel in the fin clip and the percentage of reads showing an indel in the sperm sample ranges from −0.158 to 0.928 across the nine sgRNAs (Additional file 3: Figure S1a), however this may be misleading due to differences in efficiency between the sgRNAs. Importantly, the frequencies of specific alleles do not correlate well between the tissues (Fig. 4b-c). Indeed, 27 of the 92 samples showed variants in the fin-clip sample and no variants in the germline. In addition, of the 1150 variants identified across all samples, only 77 were shared between the fin clip and the sperm sample of the same individual. This means that it is not possible to predict which, if any, of the variants seen in the fin-clip sample will be transmitted to the next generation.

Given the high efficiency of most CRISPR sgRNAs, it is feasible to recover F1 fish carrying induced indels by screening fin-clip DNA. However, these data show that screening fin clips of G0 individuals is not predictive of the germline transmission rates or the transmitted alleles.

### Direct genotyping of F1s by KASP genotyping

Another important benefit of directly screening the germline of potential founders is that it allows us to know exactly which alleles will be transmitted to the next generation, removing the need to sequence F1 individuals. We can design KASP genotyping assays (LGC Genomics) for the transmitted alleles in advance of the F1 individuals being old enough to cross. This allows us to rapidly screen F1 individuals for the transmitted alleles that they carry by simple fin clipping and genotyping PCR. Figure 4d shows the frequencies of variants called from sperm sequencing data plotted against the frequencies of carriers identified in F1 individuals by KASP for sgRNAs that have been taken through the complete pipeline. Variant frequencies called from amplicon sequencing correlate well with the number of carriers identified in F1 offspring. This provides confirmation that our indel calling is working well and allows us to use the frequencies reported from amplicon sequencing as a guide as to which sperm samples to select for IVF to generate the most F1 carriers.

Another different workflow would be to screen F1 embryos from G0 incrosses by sequencing to identify high transmitting pairs, which can then be recrossed. Indeed, similar results to ours have been reported using this scheme by Varshney et al [34]. They showed that only 3.8 % (99/2618) of somatic mutations identified in fin clips were transmitted to the F1 generation. Screening F1 embryos may be a preferable workflow for labs that do not routinely cryopreserve sperm although sperm samples can be screened without having to cryopreserve them. However, screening F1 embryos requires keeping living fish housed as pairs until the analysis is completed and then for a second cross to be carried out following analysis. Screening G0 sperm is a streamlined workflow that involves less fish work to identify loss-of-function alleles.

### GGN21GG sgRNAs have reduced efficiency compared to GGN19GG ones

**Fig. 5:**
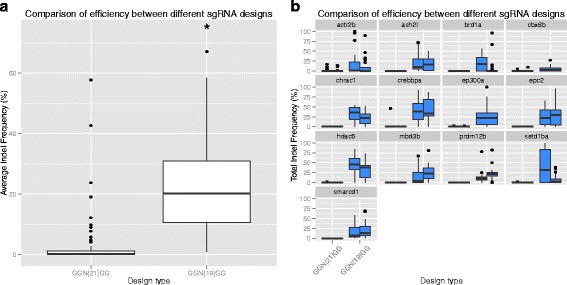
Efficiency of longer sgRNAs. **a** Longer sgRNAs (GGN21GG) tend to be less efficient than those designed to the usual consensus (GGN19GG). Plot shows the distribution of the mean indel frequency for sgRNAs with different design strategies. **b** Plot showing the distribution of induced indel frequencies for individual sgRNAs with different design strategies

Using this system we have designed and screened the efficiency of 90 sgRNAs designed to 45 genes (two per gene). These sgRNAs were designed as pairs as detailed above. After screening all 90 sgRNAs for efficiency we could see that a large proportion of them had very low activity. To investigate whether the GGN21GG design was the cause of this, we redesigned the lowest efficiency sgRNAs as single GGN19GG sites. As shown in Fig. 5, these sgRNAs are significantly more active (Wilcoxon rank sum test with continuity correction, P < 2.2 x 10^−16^). There are multiple possible explanations for this. First, the increased length of the sgRNA may affect efficiency. Second, mismatches between the 5′ Gs and the genomic sequence may reduce cutting efficiency. Thirdly, it is possible that the difference is due to the different target sites selected for each of the designs.

We think that this last possibility is unlikely given the effect size and number of sgRNAs tested. It has previously been reported that the Cas9 system can tolerate mismatches in the 5′ end of the sgRNA [1, 3, 44]. Interestingly, it has been reported in human systems that adding extra G nucleotides to the 5′ of the sgRNA reduces off-target cutting [45]. Cho et al. compared on- and off-target cutting efficiency for three different target sites using either GGN21GG or GN20GG for the design. All three targets showed decrease off-target cutting efficiency. However, for two of the target sites, the GGN20GG designs were also significantly less active at the on-target site than the corresponding GN20GG design.

Therefore, we favour the idea that it is the increased length causing the decreased efficiency. Another group [34] has also recently reported reduced cutting efficiency with the same GGN21GG design compared to GGN19GG.

## Conclusions

We have developed a system for designing and analysing CRISPR Cas9 sgRNAs in batch. The Crispr package is freely available and can be downloaded from GitHub (https://github.com/richysix/Crispr). We have developed an efficient germline screening platform using NGS. We have used this system to compare the variants found in the germline and somatic tissues of founder individuals and have shown that, in zebrafish, somatic tissues are not predictive of the alleles (or their frequencies) that are transmitted to the next generation. We have also observed a decrease in efficiency of sgRNAs designed using a different consensus sequence. It would also be possible to extend our method to screen multiple amplicons in the same samples, allowing screening of injections of multiplexed sgRNAs, as well as using it to screen samples for precise genomic changes rather than indels.

## Methods

### Crispr package

Instructions for how to use the Crispr package can be found at github.com/richysix/Crispr/. Details on the bioinformatics pipeline are supplied as a tutorial in Additional file 2.

### Design of sgRNAs

The sgRNAs used in this study were designed using both the crispr_pairs_for_deletions.pl and find_and_score_crispr_sites.pl scripts from the Crispr package. The first 90 sgRNAs were designed as pairs with a minimum separation of 30 bp and a maximum of 100 with a target consensus of N21GG. Two G bases were then added to the 5′ end of the oligos to produce the sgRNAs using T7 RNA polymerase. Low efficiency sgRNA pairs were redesigned as single guides using a target consensus of GGN19GG.

### Production of sgRNAs

To generate templates for sgRNA transcription we used the method of Gagnon et al. [5]. Target-specific oligonucleotides containing the T7 promoter sequence, the target site without the PAM, and a complementary region were annealed to a constant oligonucleotide encoding the reverse complement of the tracrRNA. The ssDNA overhangs were filled in with T4 DNA polymerase (NEB), and the resulting sgRNA template were purified using Qiaquick columns (Qiagen). We used MegaShortScript T7 kit (Life Technologies) to synthesise sgRNAs. All sgRNAs were then DNase treated and precipitated with ammonium acetate/ethanol. Cas9 mRNA was transcribed from linearised pCS2- nls-zCas9-nls plasmid using mMessage Machine SP6 kit (Life Technologies), DNase treated, and purified by phenol–chloroform extraction and EtOH precipitation. RNA concentration was quantified using Qubit spectrophotometer and diluted to 100 ng/ul (sgRNAs) or 500 ng/ul (Cas9 mRNA).

### Zebrafish husbandry

Zebrafish were maintained in accordance with UK Home Office regulations, UK Animals (Scientific Procedures) Act 1986, under project licence 70/7606, which was reviewed by the Wellcome Trust Sanger Institute Ethical Review Committee. Embryos were obtained either through natural matings or *in vitro* fertilisation and maintained in an incubator at 28.5 °C up to 5 days post fertilisation (dpf).

### MicroInjection

Approximately 1 nl total volume of 10 ng/ul (sgRNAs) and 200 ng/ul (Cas9 mRNA) was injected into the cell of one-cell stage embryos. We routinely inject 150–200 embryos per CRISPR sgRNA, raise 100 embryos and MiSeq screen 24 embryos at 24–48 h post fertilization (hpf).

### Cryopreservation of alleles

Sperm samples from G0 sgRNA-injected males were cryopreserved as previously described [46]. We usually archive sperm from 12 individuals if available. Sperm samples are split into three; two are frozen and the third is used for screening by amplicon sequencing.

### Screening by amplicon sequencing

#### Illumina library prep

To detect indels in F0 embryos, 22 injected embryos and 2 non-injected embryos were individually lysed at 24–48 hpf by Hot Shot method [47]. To detect indels in fin clips and sperm, samples were lysed using DNA extraction buffer (100 mM Tris pH 8.2, 5 mM EDTA, 200 mM NaCl, 0.2 % SDS, 100 μg/ml proteinase K) overnight at 55 °C, followed by heat inactivation of proteinase K at 80 °C for 30 min and isopropanol precipitation.

The Crispr package was used to design screening primers around each CRISPR target site. The amplicons were fixed at 250–300 bp and the sgRNA site was always offset so the sequencing efficiently covers it. The software designs nested primers in the first PCR. The second (internal) set of PCR primers have partial Illumina adaptor sequence at the 5′ end, so that the product from the second PCR can be re-amplified with full-length Illumina adaptor primers (barcoded if required). We used a set of 384 barcoded Illumina adaptor primers in the third PCR.

PCR amplifications were performed with KOD Hot Start DNA Polymerase (Novagen) following the manual and Touch down (−0.5 °C/cycle) PCR conditions: 95 °C 2 min; 18 cycles 95 °C 20 s, 65 °C → 56 °C 20 s, 70 °C 20 s; 30 cycles 95 °C 20 s, 56 °C 20 s, 70 °C 20 s; 70 °C 1 min. PCRs were checked by gel electrophoresis for the right amplicon size and the third PCRs were pooled and run on a 150 bp paired-end Miseq sequencing run.

#### Variant calling

Amplicon analysis was performed using the count_indel_reads_from_bam.pl script from the Crispr package. Before running the script, the reads are split into individual sample FASTQ files based on the barcodes. The reads were then trimmed to remove adaptor contamination and primer sequence using cutadapt [41]. Reads were filtered post-trimming to remove any reads trimmed to smaller than 50 bp. Trimmed and filtered reads were mapped to the zebrafish genome (Zv9 assembly; [48]) using BBMap (sourceforge.net/projects/bbmap/*)*. The resulting BAM files were used as the input files for count_indel_reads_from_bam.pl. The script requires a YAML configuration file detailing which BAM files to analyse for which amplicons/sgRNAs. These were produced from an instance of the Crispr package MySQL database, but could be produced by hand. The data for the sperm vs fin-clip anlaysis are in Additional files 4 and 5. The output files from the efficiency screening were collated into a single file (Additional file 6: fig_5_data.tsv) for subsequent analysis.

### KASP genotyping

Genotyping of potential F1 carriers was performed using the competitive allele-specific PCR (KASP) system (LGC Genomics Ltd. Hoddesdon, UK; http://www.lgcgroup.com/products/kasp-genotyping-chemistry) on fin-clip biopsies as previously described [46].

### Statistical analysis

Data analysis was carried out using R v3.1.3 [49]. Plots were created using ggplot 1.01 [50]. The data and code to produce the plots is provided in Additional files 4, 5, 6 and 7.

## Availability of supporting data

All sequence data has been deposited in the European Nucleotide Archive (ENA). The accession numbers are listed in Additional file 8.

## Additional files

Additional file 1: Table of CRISPR target sequences. (TXT 7 kb) Additional file 2: Tutorial for the bioinformatics including sgRNA/primer deisgn and MiSeq analysis. (PDF 7778 kb) Additional file 3: Figure S1 and Figure S2. Comparison of induced indels between germline and somatic tissues. (a) Plot shows the same data as Fig. 4a, split into separate plots for each sgRNA. (b) Plot of frequencies for individual variants in sperm versus fin clip (same data as in Fig. 4b with full axes). (PDF 374 kb) Additional file 4: Amplicon screening data used for Fig. 4a-c. (TXT 142 kb) Additional file 5: Results from KASP genotyping used for Fig. 4d. (TXT 910 bytes) Additional file 6: Amplicon screening data used for Fig. 5. (TXT 3213 kb) Additional file 7: Code used to produce the plot in Figs. 4 and 5, Additional file 3: Figure S1. (TXT 14 kb) Additional file 8: Sample accession numbers. (TSV 80 kb)

## Abbreviations

bp: base pair
CRISPR: clustered regularly interspaced short palindromic repeat
crRNA: CRISPR RNA
dpf: days post fertilization
hpf: hours post fertilization
indel: insertion/deletion
IVF: in vitro fertilisation
NGS: next generation sequencing
PAM: proto-spacer adjacent motif
sgRNA: synthetic guide RNA
tracrRNA: trans-activating RNA
