# Supplementary material for: Efficient identification of CRISPR/Cas9-induced insertions/deletions by direct germline screening in zebrafish
Source: BMC Genomics. 2016 Mar 24;17:259. doi: 10.1186/s12864-016-2563-z (PMC4806435; doi:10.1186/s12864-016-2563-z)
Supplement: Additional file 2: — Tutorial for the bioinformatics including sgRNA/primer deisgn and MiSeq analysis. (PDF 7778 kb) [file 12864_2016_2563_MOESM2_ESM.pdf]

Tutorial

The assumed workflow is

1. select CRISPR target sites for a list of targets
    - `find_and_score_crispr_sites.pl`
    - `crispr_pairs_for_deletions.pl`
2. design PCR primers for screening CRISPR cutting efficiency
    - `design_pcr_primers_for_illumina_screening.pl`
3. Analyse MiSeq sequencing of amplicons
    - `count_indel_reads_from_bam.pl`

The steps are independent of each other and do not have to all be run.
At each step there are also scripts to add the information to an SQL database. The database can also be use to automate the analysis of amplicon sequencing. See the **SQL database** section for more details.

CRISPR guide RNA design

The CRISPR design scripts scan target regions for valid CRISPR target sites and score off-target potential by mapping the CRISPR target sequence back to the target genome allowing mismatches (up to 4 at the moment). The default CRISPR target sequence to search for is N{21}GG, but this can altered As the scripts use the Ensembl database, a target region can be an Ensembl gene, transcript or exon id or the coordinates of a genomic region. At the moment, there isn't support for searching arbitrary fasta files. The input must be tab-separated
Columns are: TARGETS [REQUESTOR] [GENE_ID]

TARGETS: Acceptable targets are Ensembl exon ids, gene ids, transcript ids or genomic positions/regions. RNA Seq gene/transcript ids are also accepted. All four types can be present in one file.

REQUESTOR: This is optional. We use it for tracking purposes. The option --requestor can be used to set a requestor name for all targets and then the input file doesn't need them. It is required if you are using the SQL database to store guide RNAs as the targets are indexed by target name and requestor. Each target can have a different requestor if supplied in the input rather than by the --requestor option.

GENE_ID: Optionally an Ensembl gene id can be supplied for genomic regions.

This input can also be supplied on STDIN rather than a file.

An example command is shown below

```
# make a targets file
echo -e "ENSDARE00001194351
ENSDART00000158694
ENSDARG00000101846
5:2443741-2444279:1" > targets_file.txt

# run design script
find_and_score_crispr_sites.pl \
--target_genome /path/to/genome/file.fa \
--annotation_file /path/to/annotation/file.gff \
--target_sequence GGNNNNNNNNNNNNNNNNNNNGG \
--species zebrafish --requestor crispr_test \
targets_file.txt > crRNAs-scored.txt

# a quick description of options available
find_and_score_crispr_sites.pl --help

# for a full description of all available options
find_and_score_crispr_sites.pl --man
```

The target genome must be indexed by bwa. The appropriate genome file can be downloaded from Ensembl.
e.g.

```
# download genome file
wget ftp://ftp.ensembl.org/pub/release-81/fasta/danio_rerio/dna/Danio_rerio.GRCz10.dna.toplevel.fa.gz
gunzip Danio_rerio.GRCz10.dna.toplevel.fa.gz
# index with bwa
bwa index -a bwtsw Danio_rerio.GRCz10.dna.toplevel.fa
```

Off-targets are scored based on whether they are found in exons, introns or between genes. To do this the script requires a file of gene annotation in gff format. A helper script (dump_exons_and_introns.pl) is included to download annotation from the Ensembl database. The annotation depends on which gene build it is from. Please make sure you are using the correct version of the Ensembl API. e.g.

```
# download annotation for zebrafish from Ensembl
perl scripts/dump_exons_and_introns.pl zebrafish
```

There is also a --no_db option which stops the script connecting to the Ensembl database. It uses the supplied genome file to search for CRISPR target sites, but can therefore only accept genomic regions as input rather then Ensembl ids.

It is also possible to supply the script with a vcf file of known variants (using the --variation_file option) to avoid in the designs. Any CRISPR target site that overlaps any of the supplied variants is discarded.

Screening by amplicon sequencing - Primer Design

The `design_pcr_primers_for_illumina_screening.pl` script can be used to design nested primer pairs for amplifying regions around CRISPR target sites in order to assess the effectiveness of a CRISPR guide RNA. The amplicons produced can be analysed by sequencing or other methods such as T7 endonuclease I assay or the loss of overlapping restriction enzyme sites.

The input to the script is tab-separated
CRISPR_ID [SPECIES]

CRISPR_ids are like the ones output by the guide RNA design script. They are of the form crRNA:CHR:START-END:STRAND (e.g. crRNA:15:1001-1023:-1). If all the input CRISPR target sites are from the same species you can leave that column out of the input and supply it to the script with the --species option. It is also possible to supply ids for pairs of gRNAs in the form crRNA:15:1001-1023:-1.crRNA:15:1051-1073:1

The script retrieves sequence around the CRISPR target sites and uses it to design nested pairs of primers. By default, the internal pairs have partial Illumina adaptor sequence added to allow the creation of sequencing-ready libraries. This can be altered using the --left_adaptor/right_adaptor options.

The allowed product sizes of the amplicons can be altered using the --ext_product_size and --int_product_size options.
The default sizes are:
Ext: 300-600
Int: 250-300

The internal size is because we use 150 bp paired-end MiSeq reads. The script tries to make one end of the amplicon a reasonable distance from the CRISPR target site. You need to allow enough room between the primer and target site to allow for bigger deletions to be detected. This distance can be set with the --target_offset option.

By default, the script also searches the amplicon sequence for unique restriction enzyme sites that overlap the CRISPR target site that can be used to assess cutting efficiency. This can be turn off with the --norestriction_enzymes option. This behaviour may change in future releases. (i.e. the default behaviour may change to not checking restriction sites).

An example command is shown below

```
# make a crRNA file
echo -e "crRNA:5:2443696-2443718:1
crRNA:5:2468559-2468581:-1
crRNA:5:2435853-2435875:-1
crRNA:5:2443844-2443866:-1" > crRNA_file.txt

# run design script
design_pcr_primers_for_illumina_screening.pl \
--species zebrafish --norestriction_enzymes \
--primer3file /path/to/config/primer3.cfg \
--output_file miseq_primers.tsv crRNA_file.txt

# a quick description of options available
design_pcr_primers_for_illumina_screening.pl --help

# for a full description of all available options
design_pcr_primers_for_illumina_screening.pl --man
```

Output

The script outputs a file containing the designed primer sequences. This file is tab-separated with the following columns

```
crispr_pair_name                            This is for pairs of guide RNAs. NULL if input is a single
CRISPR_ID
crRNA_name                                  name of the guide RNA (crRNA:CHR:START-END:STRAND)
ext_primer_pair_id                          name for the external amplicon (CHR:START-END:STRAND)
left_ext_primer_id                          name for the left external primer
left_ext_primer_seq                         sequence for the left external primer
right_ext_primer_id                         name for the right external primer
right_ext_primer_seq                        sequence for the right external primer
int_primer_pair_id                          name for the internal amplicon (CHR:START-END:STRAND)
left_int_primer_id                          name for the left internal primer
left_int_primer_seq                         sequence for the left internal primer
right_int_primer_id                         name for the right internal primer
right_int_primer_seq                        sequence for the right internal primer
int-illumina_tailed_primer_pair_id          same as int primers but with partial illumina adaptor on
left_int-illumina_tailed_primer_id          same as int primers but with partial illumina adaptor on
left_int-illumina_tailed_primer_seq         same as int primers but with partial illumina adaptor on
right_int-illumina_tailed_primer_id         same as int primers but with partial illumina adaptor on
right_int-illumina_tailed_primer_seq        same as int primers but with partial illumina adaptor on
ext_sizes                                   size of external amplicon
int_sizes                                   size of internal amplicon
```

We order just the external and internal-illumina_adaptor primers, but the internal primers are included on their own in the output for completeness.

Screening by amplicon sequencing - Analysis of sequencing

The output of our sequencing is a set of fastq files, one for each sample barcode. The example commands in the next sections show a representative command for a single barcode.

Trim Adaptors

We remove contaminating adaptor sequence using cutadapt http://cutadapt.readthedocs.org/en/latest/guide.html.

```
# trim adaptor sequence
mkdir trimmed
cutadapt \
--anywhere ILLUMINA-ADAPTOR-1=AATGATACGGCGACCACCGAGATCTACACTCTTTCCCTACACGACGCTCTTCCGATCT \
--anywhere ILLUMINA-ADAPTOR-2=AGATCGGAAGAGCGTCGTGTAGGGAAAGAGTGTAGATCTCGGTGGTCGCCGTATCATT \
--anywhere ILLUMINA-ADAPTOR-3=GAGATCGGTCTCGGCATTCCTGCTGAACCGCTCTTCCGATCT \
--anywhere ILLUMINA-ADAPTOR-4=AGATCGGAAGAGCGGTTCAGCAGGAATGCCGAGACCGATCTC \
-e 0.12 -q 20 -n 6 --overlap=10 \
-o trimmed/15708_1#1.trim.fastq \
--info-file trimmed/15708_1#1.trim.info \
fastq/15708_1#187.fastq > trimmed/15708_1#187.trim.o

# filter reads under 50 bp
filter_fastq.pl \
--interleaved trimmed/15708_1#1.trim.fastq
# output file is trimmed/15708_1#1.trim.filt.fastq
```

Map

The trimmed reads are mapped to the genome using BBMap http://sourceforge.net/projects/bbmap/. It is able to effectively map reads containing large deletions. The genome first needs to be indexed by BBMap.

```
# index genome file
java -ea -Xmx20g -cp bbmap/current align2.BBMap \
ref=Danio_rerio.GRCz10.dna.toplevel.fa \
path=genomes/bbmap/ build=10 midpad=100000

# map
mkdir mapped
java -ea -Xmx12g -Xms12g -cp bbmap/current align2.BBMap \
path=genomes/bbmap build=10 \
in=trimmed/15708_1#1.trim.filt.fastq \
out=mapped/15708_1#1.sam threads=8

# convert sam to bam and sort
samtools view -hbSF 2048 mapped/15708_1#1.sam | samtools sort - 15708_1#1
```

Call indels

The indel calling script `count_indel_reads_from_bam.pl` is designed to call indels from an entire sequencing run. The script requires a configuration file in YAML http://www.yaml.org/spec/1.2/spec.html format. An example is shown below

```
---
name: miseq_15708
run_id: 15708
lane: 1
plates:
  -
    name: 1
    wells:
      -
        well_ids: A01,A02,A03,A04,A05,A06,A07,A08,A09,A10
        indices: 1,2,3,4,5,6,7,8,9,10
        sample_names: 187_1,187_2,187_3,187_4,187_5,187_6,187_7,187_8,187_9,187_10
        plexes:
          -
            name: 187
            region_info:
              -
                crisprs:
                  - crRNA:15:970-992:-1
                gene_name: gene_1
                region: 15:890-1160:1
              -
                crisprs:
                  - crRNA:21:20100501-20100523:1
                gene_name: gene_2
                region: 21:20100435-20100700:1
```

The script is able to call indels in multiple regions allowing gRNAs to be used in multiplex. The above file shows 10 samples labelled with barcodes 1-10 in wells A01-A10 to be analysed in 2 different regions. We routinely run 4 plates worth of samples on a single run. The samples are divided into sets (subplex) that are all to be analysed for the same amplicons.

```
mkdir results
count_indel_reads_from_bam.pl \
--ref Danio_rerio.GRCz10.dna.toplevel.fa \
--sample_dir mapped --no_pindel \
--pc_filter 0.01 --consensus_filter 50 \
--verbose --output_dir results --output_file 15708.txt \
--dindel_scripts /path/to/packages/dindel-python \
--dindel_bin /path/to/bin/dindel \
15708.yml

# a quick description of options available
count_indel_reads_from_bam.pl --help

# for a full description of all available options
count_indel_reads_from_bam.pl --man
```

The script uses dindel to call indels so this must be installed and either in the current path or you can supply the path to it using the --dindel_bin option Since dindel was designed to call indels in non-mosaic situations from reasonably low coverage data the script first assesses which reads contain an indel, downsamples them and outputs them to separate bam files which are then given to dindel to call the indels. If a variant overlaps more than one CRISPR target site it is designated as type crispr_pair and will be reported for each site that it overlaps.

Filtering

There are a set of options that are used to filter candidate indels.

--overlap_threshold
Only indels that overlap the supplied CRISPR target site are kept. This sets the distance from the predicted cut-site that a variant must overlap to be counted. default: 10

--pc_filter
This is the threshold for the percentage of reads that a variant has to reach to be output. This is to avoid inclusion of sequencing and PCR errors which tend to be at much lower levels than true variants. default: 0.01

--consensus_filter
This is the threshold for the length of the consensus sequence for the reads that support a variant. The default setting is an attempt to avoid counting primer-dimer which can be a significant problem in some cases. default: 50

--low_coverage_filter
This turns on a filter to discard samples that fall below an absolute number of reads to avoid samples with low numbers of reads. If this option is not set, all samples are processed. This option can be suppied with or without a number. Without a number filtering is turned on at the default level. default: 100

--low_coverage_per_variant_filter
This turns on a filter to discard individual variants that fall below an absolute number of reads. If this option is not set, variants are filtered by percentage only. This option can be suppied with or without a number. Without a number filtering is turned on at the default level. default: 10

Results

The output file from `count_indel_reads_from_bam.pl` contains the following columns:

```
plex                            name from the YAML file
plate                           plate number
subplex                         name of the analysis set
well                            well id
sample_name                     name of the sample from the YAML file
gene_name                       gene name for the analysis
group_name                      the group to which this variant has been allocated. Used for the
visualisations: see below.
amplicon                        region analysed
caller                          name of caller (DINDEL/CIGAR/PINDEL)
type                            crispr or crispr_pair
crispr_name                     name of CRISPR target site
chr                             chromosome
variant_position                starting position of the called variant. This is the base before the
deletion/insertion
reference_allele                Reference allele in vcf
alternate_allele                Variant allele in vcf
num_reads_with_indel            number of reads that contain this indel
total_reads                     number of reads covering the region in that sample
percentage_reads_with_indel     num_reads_with_indel/total_reads
consensus_start                 start position of the consensus sequences
ref_seq                         Reference consensus
consensus_alt_seq               Variant consensus
```

The variants are reported in vcf format
e.g.

```
10   456   GATCT    G     - deletion of ATCT
10   462   T        TAG   - insertion of AG
10   462   TAT      TC    - complex indel. deletion of AT plus insertion of C
```

The output also contains consensus sequences for the reference and variant to allow manual inspection of the variant.

```
# example line of output
miseq_15708  1  187  A02  187_2  gene_1  1  15:900-1160:1  DINDEL  crispr  crRNA:15:970-992:-1  15  972
GTGAG  G  4896  73173  0.0669099257923004
TTTAGTTTAATTAAAGAGCTTTTCAAAATAAATTGCTGAATTAAAATAAAGTATTGACCGTGAGTCCCGCAGTCGAGGAGAGAACGTTCATTATTTTGAACAC
ATTTAAGAAAATGAAGGATATTAG
TTTAGTTTAATTAAAGAGCTTTTCAAAATAAATTGCTGAATTAAAATAAAGTATTGACCGTCCCGCAGTCGAGGAGAGAACGTTCATTATTTTGAACACATTT
AAGAAAATGAAGGATATTAG

# The consensus sequences can be used to check the alignment and variant call
TTTAGTTTAATTAAAGAGCTTTTCAAAATAAATTGCTGAATTAAAATAAAGTATTGACCGTGAGTCCCGCAGTCGAGGAGAGAACGTTCATTATTTTGAACAC
ATTTAAGAAAATGAAGGATATTAG
TTTAGTTTAATTAAAGAGCTTTTCAAAATAAATTGCTGAATTAAAATAAAGTATTGACCG----
TCCCGCAGTCGAGGAGAGAACGTTCATTATTTTGAACACATTTAAGAAAATGAAGGATATTAG
```

Visualisations

To help display the results, there are 2 R scripts that produce visualisations of the data These require R to be installed.

`crispr_results_tile_plots.R`

This script takes the output of `count_indel_reads_from_bam.pl` and produces a series of plate plots showing, for each well, the total percentage of reads containing an indel and the total number of reads covering the region.

```
crispr_results_tile_plots.R -d results \
--scripts_directory=/path/to/Crispr/scripts/ --plate_type=96 \
--basename=15708 15708.txt
```

`variant_display.R`

This script produces diagrams showing the indels within the samples. Deletions are shown as a gap in the line and insertions as shown in red.

```
variant_display.R -d results \
--display_type=pdf --basename=15708 15708.txt
```

Scripts

As well as the main design and analysis scripts, there are a number of accessory scripts which are detailed below. For more information use the --help or --man options for each individual script.

filter_fastq.pl

This is a simple script to filter fastq files after reads have been trimmed. It discards reads/read pairs where one of the reads is shorter than the --length_threshold option [default=40]

```
filter_fastq.pl \
--length_threshold 60 --interleaved 15708_1#1.trim.fastq
```

score_crisprs_from_id.pl

Accessory script to score CRISPR target sites from a crispr name of the form crRNA:CHR:START-END:STRAND

```
score_crisprs_from_id.pl \
--target_genome /path/to/genome/file.fa \
--annotation_file /path/to/annotation/file.gff \
--target_sequence GGNNNNNNNNNNNNNNNNNNNGG \
--singles --species zebrafish crRNA_file.txt > crRNAs-scored.txt
```

SQL database

The SQL database is designed to hold information on CRISPR target sites/guide RNAs including construction oligos and PCR primers for screening. As well as this, it has tables to store the results of amplicon sequencing including the variants found and KASP genotyping assays. Also, if the database is loaded with information on samples and guide RNAs etc. it can be used to automate the analysis pipeline.

The tables in the database are:

```
target                      cas9
plate                       cas9_prep
crRNA                       injection
crRNA_pair                  injection_pool
coding_scores               sample
off_target_info             plex
plasmid_backbone            analysis
construction_oligos         analysis_information
expression_construct        sequencing_results
guideRNA_prep               allele
primer                      allele_to_crispr
primer_pair                 sample_allele
amplicon_to_crRNA           kasp
enzyme
enzyme_ordering
restriction_enzymes
```

Target table

A Target is a stretch of DNA that can be associated with CRISPR targets.

target_id	target_name	assembly	chr	start	end	strand	species	requires_enzyme	ge
257	ENSDARE00000322522	Zv9	21	18273817	18274310	1	zebrafish	y	ENSDARG

crRNA/crRNA_pair

A crRNA represents a CRISPR target site and is linked to a particular target and requestor. crRNAs can be paired and this is stored in the crRNA_pair table.

crRNA_id	crRNA_name	chr	start	end	strand	sequence	num_five_prime_Gs
436	crRNA:21:18273990-18274012:1	21	18273990	18274012	1	TTGGAGTGGTGGAGCCTCCGAGG	0

Tables coding_scores, off_target_info, plasmid_backbone, construction_oligos and expression_construct hold other information about CRISPR target sites.

guideRNA_prep

A guideRNA_prep is a particular preparation (protein/RNA) of an sgRNA. The table holds information about the date it was made and who made it.

guideRNA_prep_id	crRNA_id	guideRNA_type	concentration	made_by	date	plate_id	well_id
1	242	sgRNA	0.0	cr_user2	2014-01-01	NULL	NULL

primers

The primer, primer_pair and amplicon_to_crRNA tables hold information about screening primers and which ones are for which CRISPR targets.

primer

primer_id	primer_sequence	primer_chr	primer_start	primer_end	primer_strand	primer_tail	plate_i
1	CCAATATAGTGCTCCACATCTGTTACA	23	27843893	27843919	1	NULL	5

primer_pair

primer_pair_id	type	left_primer_id	right_primer_id	chr	start	end	strand	product_size
1	int-illumina	1	46	23	27843893	27844076	1	184

amplicon_to_crRNA

primer_pair_id	crRNA_id
1	1

EnzymeInfo

The enzyme tables store information on unique restriction sites near CRISPR target sites.

Cas9

The type of Cas9 construct and type of prep (rna vs protein) are stored in the cas9 and cas9_prep tables.

cas9

cas9_id	name	type	vector	species
1	pCS2-ZfnCas9n	ZfnCas9n	pCS2	s_pyognenes

cas9_prep

cas9_prep_id	cas9_id	prep_type	made_by	date	notes
113	1	rna	cr_user2	2014-04-13	M113

Injections

The injection and injection_pool tables were designed to hold information on sgRNAs injected into zebrafish but can be used for other things such as other species or transfections.

injection

injection_id	injection_name	cas9_prep_id	cas9_concentration	date	line_injected	line_raised	sorted_by
1	49	1	200.0	2014-02-05	H0001	MR0001	NULL

injection_pool

injection_id	crRNA_id	guideRNA_prep_id	guideRNA_concentration
6	501	6	10
6	502	7	10

Samples

A sample is a preparation of DNA from cells that have been injected/transfected with sgRNA(s) to be analysed.

sample_id	sample_name	sample_number	injection_id	generation	type	species
1	49_1	1	1	G0	sperm	zebrafish

Sequencing Information

plex

A plex represents a multiplexed sequencing run.

plex_id	plex_name	run_id	analysis_started	analysis_finished
1	mpx22	15524	2015-02-23	NULL

Within a sequencing run, an Analysis is a set of samples that are all sequenced for the same amplicons. The analysis and analysis_information tables hold the information on the amplicons and sgRNAs in an Analysis.

analysis

analysis_id	plex_id	analysis_started	analysis_finished
1	4	2014-06-09	NULL

analysis_information

analysis_id	sample_id	primer_pair_id	barcode_id	plate_number	well_id
1	619	46	1	1	A01

sequencing_results, allele, allele_to_crispr, sample_allele and kasp hold the results of the analysis including genotyping assays.

sequencing_results

sample_id	crRNA_id	fail	num_indels	total_percentage_of_reads	percentage_major_variant	total_reads
1	1	0	5	0.2456	0.0853	23753

allele

allele_id	chr	pos	ref_allele	alt_allele	ref_seq
1	23	25637957	AGCTTG	A	GACTAGACTAGATCATATGACAGATCGACGATACGATACGTACGATACGATAGCTTGA[illegible]

allele_to_crispr

allele_id	crRNA_id
1	1

sample_allele

sample_id	allele_id	percentage_of_reads
1	1	0.0853

kasp

kasp_id	allele_id	allele_number	allele_specific_primer_1	allele_specific_primer_2	common_primer_1
1	1	sa30756	GCAGAGAGAAGGAAGCCGAGA	CAGAGAGAAGGAAGCCGAGG	ATCTGGTGTGTCAGGCTGGGT[illegible]

In order to use the database to automate analysis the following tables need to be used:
target, crRNA, guideRNA_prep, primer, primer_pair, amplicon_to_crRNA, cas9, cas9_prep, injection, injection_pool, sample, plex, analysis and analysis_information

The full schema of the database can be found in `sql/schema_mysql.sql` or `sql/schema_sqlite.sql`. The connection settings for the database can be set either by supplying a config file or by using environment variables. The config file is tab-separated key value pairs.

```
# MySQL
driver  mysql
host    hostname
user    username
pass    pasword
port    port
dbname  databasename

# SQLite
driver  sqlite
dbname  databasename
dbfile  dbfilename
```

Otherwise, you can set the following environment variables
For MySQL: MYSQL_DBHOST, MYSQL_DBPORT, MYSQL_DBUSER, MYSQL_DBPASS, MYSQL_DBNAME
For SQLite: SQLITE_DBFILE, SQLITE_DBNAME

add_targets_to_db_from_file.pl

This is used to add information about targets (i.e. a region of DNA to search for CRISPR target sites).
It is designed to take some of the information output by the CRISPR design scripts.
The columns target_name, start, end, strand, requires_enzyme and requestor cannot be null.

```
# make targets file
head -n1 crRNAs-scored.txt | cut -f1-14 > targets-info.txt
cut -f1-14 crRNAs-scored.txt | sort -u | grep -v ^# >> targets-info.txt

# add target info to db
add_targets_to_db_from_file.pl \
--crispr_db /path/to/config.conf targets-info.txt
```

add_crRNAs_to_db_from_file.pl

This is used to add information about CRISPR target sites.
It is designed to take information output by the CRISPR design scripts.
The targets must exist in the database and the columns start, end, strand, sequence, num_five_prime_Gs, and target_id cannot be null.

```
# make crispr info file
echo "target" | cat - crRNA_file.txt | grep -f - crRNAs-scored.txt | \
cut -f2,8,12,15-31 | sed -e 's|^target|#target|' > crRNA-info.txt

# add crRNA info to db
add_crRNAs_to_db_from_file.pl \
--crispr_db /path/to/config.conf \
--plate_num 1 --plate_type 96 --fill_direction row \
--designed 2015-09-22 --construction_oligos t7_fill-in_oligos crRNA-info.txt
```

add_crispr_pairs_to_db_from_file.pl

```
# add crRNA pair info to db
add_crispr_pairs_to_db_from_file.pl \
--crispr_db /path/to/config.conf \
--plate_num 2 --plate_type 96 --fill_direction row \
--designed 2015-09-22 --construction_oligos t7_fill-in_oligos crRNA_pair-info.txt
```

add_primer_pair_plus_enzyme_info_for_crRNAs_to_db_from_file.pl

Script to add screening primer information. It will also add information on unique restriction sites.
The input file should contain the following columns:

- product_size - size of PCR product (Int)
- crisprs - comma-separated list of crRNAs covered by amplicon
- left_primer_info - comma-separated list (primer_name,sequence)
- right_primer_info - comma-separated list (primer_name,sequence)

Optional columns are:

- well_id - well id to use for adding primers to db.
  (A01-H12 for 96 well plates. A01-P24 for 384 well plates.)
- enzyme_info - comma-separated list of enzymes that cut the amplicon and the crispr target site uniquely
  each item should consist of Enzyme_name:Site:Distance_to_crispr_cut_site

Example

```
# make primer info files
perl -F"\t" -lane 'if($. == 1){
print "#", join("\t", qw{ crisprs left_primer_info right_primer_info product_size } ); }
else{ print join("\t", $F[1], join(q{,}, @F[3,4]), join(q{,}, @F[5,6]), $F[18], ) }' \
miseq_primers.tsv > ext_primers.tsv

perl -F"\t" -lane 'if($. == 1){
print "#", join("\t", qw{ crisprs left_primer_info right_primer_info product_size } ); }
else{ print join("\t", $F[1], join(q{,}, @F[13,14]), join(q{,}, @F[15,16]), $F[19], ) }' \
miseq_primers.tsv > int_primers.tsv

# add primers
add_primer_pair_plus_enzyme_info_for_crRNAs_to_db_from_file.pl \
--crispr_db /path/to/config.conf --type ext-illumina \
--plate_num 1 --plate_type 96 --fill_direction row ext_primers.tsv

add_primer_pair_plus_enzyme_info_for_crRNAs_to_db_from_file.pl \
--crispr_db /path/to/config.conf --type int-illumina_tailed \
--plate_num 1 --plate_type 96 --fill_direction row int_primers.tsv
```

If the option --plate_num is set a plate name of the form sprintf("CR_%06d%s", plate_num, suffix) with a suffix depending on the primer type.
e.g. --plate_num 1 --type ext-illumina would be stored in a plate named CR_000001f
--plate_num 1 --type int-illumina_tailed would be stored in a plate named CR_000001h

get_pcr_primers_from_db.pl

This script gets info about primers on a given plate and prints them to a tsv file for ordering. It can output everything on the plate or particular wells.

```
# print primers for plate 1f
echo CR_000001f | get_pcr_primers_from_db.pl \
--crispr_db /path/to/config.conf \
--well_range A01-A02 > CR_000001f.tsv
```

add_guide_RNA_preps_to_db_from_file.pl

The database has tables for both CRISPR target sites and the actual guide RNA prep that is injected/transfected. This script adds information on guide RNA preps.

```
# add guide RNA preps
add_guide_RNA_preps_to_db_from_file.pl \
--crispr_db /path/to/config.conf --plate_type 96 --fill_direction row gRNA-info.txt
```

A guide RNA prep must exist in the database in order to use the database to automate analysis. To add guide RNA preps for any CRISPR target in the db that doesn't have one use this to add dummy sgRNA preps:

MySQL

```
mysql -h $MYSQL_DBHOST -P $MYSQL_DBPORT -u $MYSQL_DBUSER -p$MYSQL_DBPASS $MYSQL_DBNAME -Bse \
"INSERT into guideRNA_prep \
SELECT NULL as guideRNA_prep_id, crRNA_id, "sgRNA" as guideRNA_type, \
0.0 as concentration, "user1" as made_by, "2014-01-01" as date, NULL as plate_id, NULL as well_id \
FROM crRNA cr \
WHERE crRNA_id NOT IN \
(SELECT crRNA_id FROM guideRNA_prep )"
```

SQLite

```
echo "INSERT into guideRNA_prep \
SELECT NULL as guideRNA_prep_id, crRNA_id, 'sgRNA' as guideRNA_type, \
0.0 as concentration, 'user1' as made_by, '2014-01-01' as date, NULL as plate_id, NULL as well_id \
FROM crRNA cr \
WHERE crRNA_id NOT IN \
(SELECT crRNA_id FROM guideRNA_prep );" | sqlite3 $SQLITE_DBFILE
```

add_cas9_preps_to_db.pl

This adds information on both Cas9 objects and Cas9Preps. If the Cas9 does not exist in the database already it is added.

```
add_cas9_preps_to_db.pl \
--crispr_db /path/to/config.conf cas9_prep-test_info.txt
```

add_injection_info_to_db_from_file.pl

An injection represents which Cas9/sgRNAs were used in a particular experiment.

```
add_injection_info_to_db_from_file.pl \
--crispr_db /path/to/config.conf  injection-test_info.txt
```

add_samples_to_db_from_sample_manifest.pl

This script adds individual samples to the database.

```
add_samples_to_db_from_sample_manifest.pl \
--crispr_db /path/to/config.conf samples-test_info.txt
```

add_analysis_information_to_db_from_file.pl

This script is used to add the information about which samples are to analysed for which amplicons/sgRNAs.

```
# add analysis info
add_analysis_information_to_db_from_file.pl \
--crispr_db /path/to/config.conf \
--plex_name miseq1 --run_id 10001 --analysis_started 2014-03-15 \
--sample_plate_format 96 --sample_plate_fill_direction row \
--barcode_plate_format 96 --barcode_plate_fill_direction row \
 analyses-test_info.txt
```

create_YAML_file_from_db.pl

This script creates the YAML file required by `count_indel_reads_from_bam.pl` from the information in the database.

```
create_YAML_file_from_db.pl \
--crispr_db /path/to/config.conf --plex miseq1
```
