# Supplementary material for: Efficient identification of CRISPR/Cas9-induced insertions/deletions by direct germline screening in zebrafish
Source: BMC Genomics. 2016 Mar 24;17:259. doi: 10.1186/s12864-016-2563-z (PMC4806435; doi:10.1186/s12864-016-2563-z)
Supplement: Additional file 3: Figure S1 and Figure S2. — Comparison of induced indels between germline and somatic tissues. (a) Plot shows the same data as Fig. 4a, split into separate plots for each sgRNA. (b) Plot of frequencies for individual variants in sperm versus fin clip (same data as in Fig. 4b with full axes). (PDF 374 kb) [file 12864_2016_2563_MOESM3_ESM.pdf]

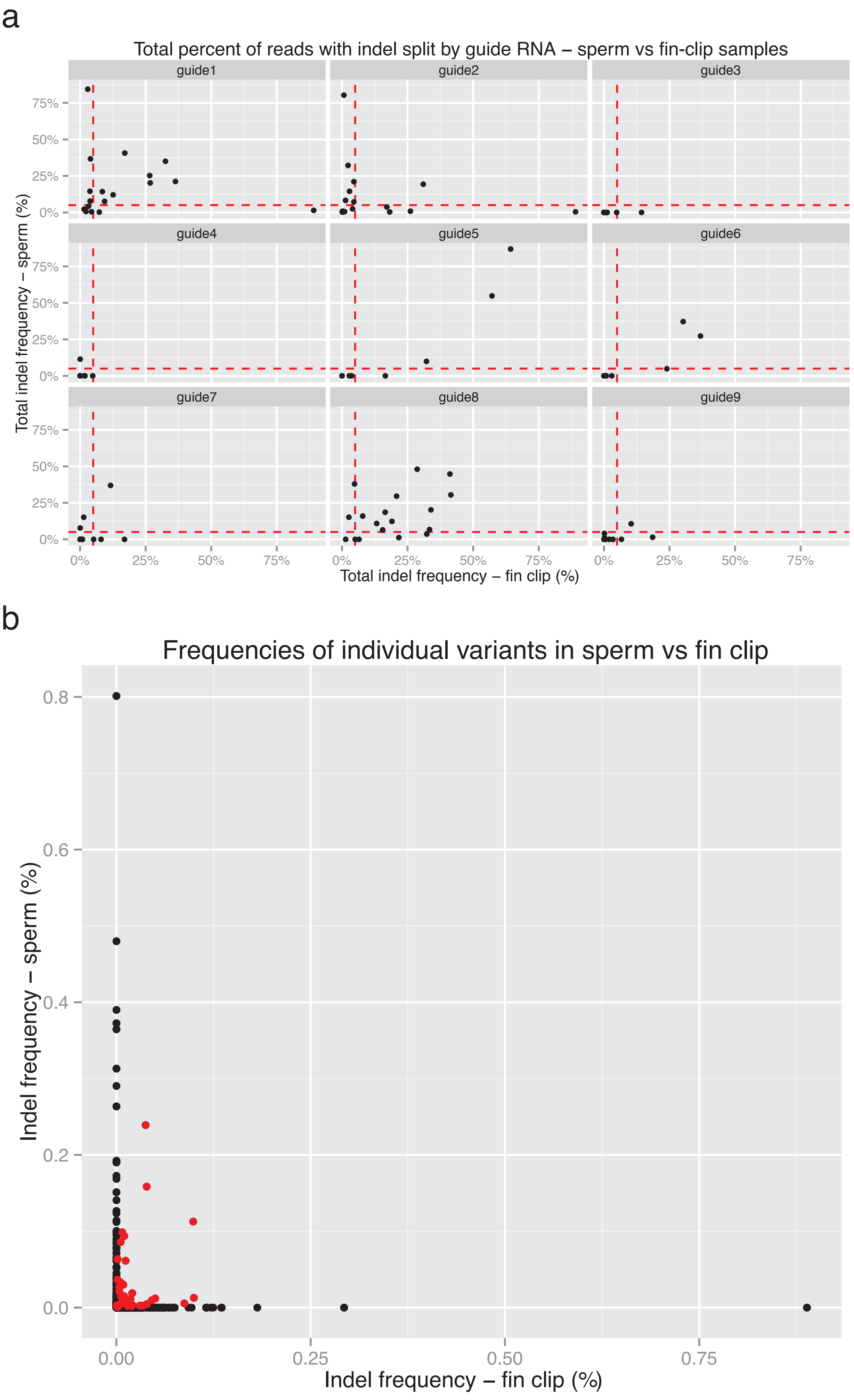
a
Total percent of reads with indel split by guide RNA – sperm vs fin-clip samples
guide1
guide2
guide3
guide4
guide5
guide6
guide7
guide8
guide9
Total indel frequency – sperm (%)
Total indel frequency – fin clip (%)
0%
25%
50%
75%
b
Frequencies of individual variants in sperm vs fin clip
Indel frequency – sperm (%)
Indel frequency – fin clip (%)
0.0
0.2
0.4
0.6
0.8
0.00
0.25
0.50
0.75
